# A Comprehensive Analysis of Robot-Assisted Surgery Uptake in the Pediatric Surgical Discipline

**DOI:** 10.3389/fsurg.2019.00009

**Published:** 2019-03-12

**Authors:** Nicolas Fernandez, Walid A. Farhat

**Affiliations:** ^1^Division of Urology, Hospital for SickKids, University of Toronto, Toronto, ON, Canada; ^2^Department of Urology, Fundación Santa Fe de Bogota, Bogota, Colombia; ^3^Division of Urology, Hospital Universitario San Ignacio, Pontificia Universidad Javeriana, Bogota, Colombia

**Keywords:** robot-assisted surgery, laparoscopy, pediatrics, urology, minimally invasive surgery, bibliometric analysis

## Abstract

**Introduction:** Robotic assisted surgery (RAS) is one of the most recent surgical approaches that has quickly been adopted by the pediatric urology community. Over the last decade, a vast amount of manuscripts has been published, supporting the safety and applicability of RAS in the pediatric population. The quality of published literature about this innovative technology remains supported by case-reports and retrospective case-series. Historical behavior of literature productivity and implementation of laparoscopy followed a similar trend. We present the historical publication uptake of RAS in pediatric urology and other surgical disciplines using a bibliometric comparison of the most cited manuscripts.

**Materials and Methods:** A systematic search and review of the literature was undertaken by the authors. Literature search was performed in OVID, PubMed, EMBASE, Scopus, Web of Science, and Google Scholar. The search period included all publications between 1985 and June 2018. All languages were included. Data analysis for graphical representation was performed using VOSviewer^®^ version 1.6.8 and Impact Index Analysis was used to adjust the citations by the time since publication.

**Results:** A total of 1,014 titles were identified. After applying exclusion criteria, 200 papers were included for the RAS arm and 402 for the laparoscopic one. Case-series was the most common type of publication. Average citations for laparoscopic manuscripts was 23 (*SD* ± 31) and for RAS was 20 (*SD* ± 31.5). The impact index analysis showed an average of 95 (*SD* ± 167) for laparoscopic manuscripts vs. 66 (*SD* ± 101) for RAS. The laparoscopic manuscript with the highest citation count had 199 citations with an impact index of 12.1. And the RAS manuscript with the highest citation count had 280 citations and an impact index of 4.3.

**Conclusion:** Literature productivity in pediatric laparoscopic and RAS has quickly grown. Pediatric Urologists play a key role in the introduction of this innovative tool. Literature supporting its implementation and future consolidation requires to focus on increasing the level of evidence.

## Introduction

Robotic surgery is one of the most recent surgical approaches that has quickly been adopted by the pediatric urology community. Since the first reported cases of laparoscopic surgery in the early 1990's and Peter's et al. first robot-assisted laparoscopic procedure on a pediatric patient in 2002, a vast amount of experience has been gained in the pediatric urological RAS field (1–3). By 2006, the application of RAS in children remained largely unexplored and the perspective of pediatric urologists was polarized (4, 5). Over the last decade, a vast amount of manuscripts have been published, supporting the safety and applicability of RAS in the pediatric population with an increase of 236.6% per year by 2016 (6).

Pediatric Urology has been the specialty that continue to lead this field with pyeloplasty being the most frequently performed procedure to date (7). More recent data support that more than 80% of pediatric urologists see a clear role for RAS in the pediatric population (5). The quick growth of RAS has been supported by the fact that surgeons can perform complex reconstructive procedures with much shorter learning curves compared to regular laparoscopy (8–10). In fact it has been seen that RAS-naïve surgeons who are performing suturing for the first time, they do it much faster than with laparoscopy (11). More recent studies are now supporting shorter lengths of stay and fewer complications for RAS cases compared to laparoscopic ones (12). Nonetheless, the quality of published literature about this innovative technology remains supported by case-reports and retrospective case-series. Historical behavior of literature productivity and implementation of laparoscopy followed a similar trend. Previous bibliometric analysis studies have shown how literature productivity and consumption (citations) cannot be interpreted the same way for all specialties in a universal way (13–15). For instance, topics like coronary artery disease, or cancer have more visibility and will be of more interest to more medical fields. This translates to higher citation counts for these publications, but specialties like pediatric RAS surgery where the amount of specialists interested in this field is smaller a proportional smaller citation count is seen. This proportion needs to be kept in mind when it comes to interpreting the citation counts based on the specialty “size”.

Based on a Progressive Scholarly Acceptance analysis, RAS has not passed the transition point yet and remains un-accepted by the scientific community (16). Innovative technologies are rarely implemented universally and RAS is not the exception (6). Limitations for RAS and laparoscopy, as innovations, in the surgical field have gone through similar paths, both technologies have had to overcome critics and prove to be safe and replicable. We hypothesize that RAS has taken off in a much faster way compared to laparoscopy, considering that RAS enables surgeons the possibility of performing complex procedures with a shorter learning curve. For these reasons, we hereby present a mathematical analysis (need to consider statistical analysis) of a literature review to show the results of a historical bibliometric comparison of the most cited manuscripts since laparoscopy and RAS were implemented.

## Methods

A systematic search and review of the literature was undertaken by the authors following the PRISMA concepts. Literature search was performed in OVID, PubMed, EMBASE, Scopus, Web of Science, and Google Scholar. A comprehensive search included the MeSh terms: Pediatrics, minimally invasive surgical procedures, laparoscopy, urology, and robotics. The search period included all publications between 1985 and June 2018. Citation count per manuscript was taken from Scopus, Web of Science and Google Scholar. A secondary search was performed following the same methodology to include pediatric surgical procedures with the MeSh terms: Pediatric Surgery, robotics. No citation analysis was performed on this secondary analysis. Otolaryngologic and Neurosurgical publications were excluded. For manuscripts with different citation counts on each database, we used the highest citation count out of the three databases. Citation data extraction was performed in a 2-day period between June 20th and 21st of 2018. Impact index analysis (IIA) was initially developed as a way to adjust the citation count interpretation based on the time since publication. For this reason, we used the IIA in the present study to interpret our results. We followed the formula reported by Fernandez et al. considering influential manuscripts with low scores (13).

All extracted titles and abstracts were screened for relevance and disagreements were resolved by consensus. Duplicated titles and abstracts that did not disclose any information about pediatric urological robot-assisted or laparoscopic surgery were excluded. For manuscripts comparing regular laparoscopy with robot-assisted surgery, the title was included in the robot-assisted analysis. All languages were included. Data analysis for graphical representation was performed using VOSviewer^®^ version 1.6.8 (http://www.vosviewer.com).

Comparisons of continuous variables was carried out with *t*-tests and ANOVA when more than two set of groups were compared. Statistical analyses were analyzed using SPSS v. 25.0 (SPSS 25.0—SPSS Inc., Chicago, Illinois). *P* <0.05 were considered statistically significant.

## Results

**Figure 1:**
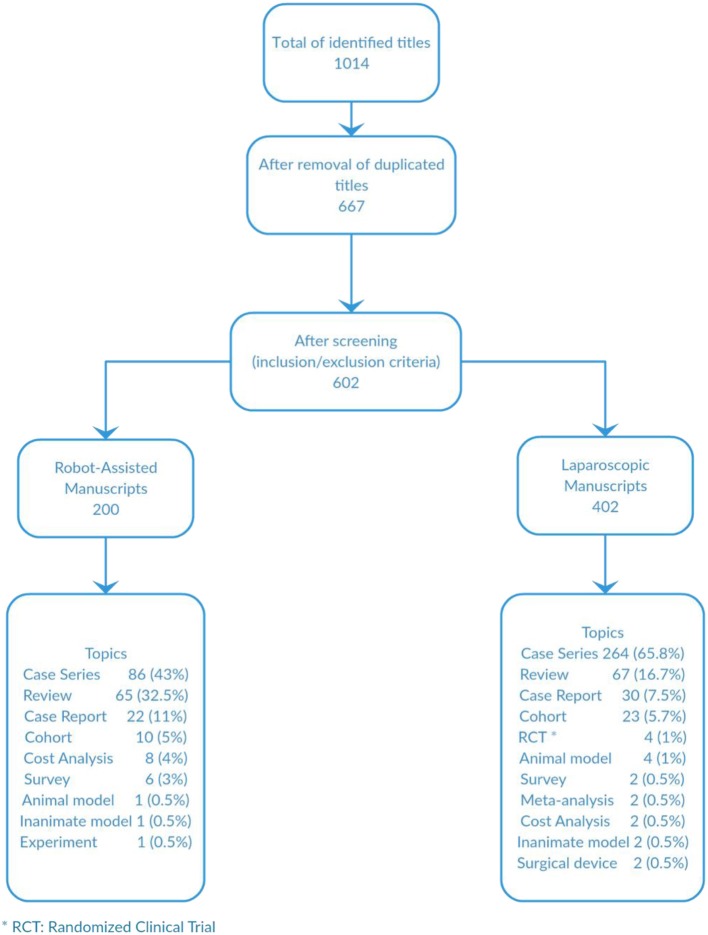
Manuscript selection and distribution of types of studies.

A total of 1,014 titles were identified. After duplicated titles were excluded, 667 titles were screened and after applying exclusion criteria, 602 titles were included for analysis; 200 for the robot-assisted arm and 402 for the laparoscopic one. Case-series was the most common type of publication for both arms followed by review articles and case reports (Figure 1). There was only one experiment with an animal model and another with an inanimate one in the RAS analysis and 4 and 6, respectively, in the laparoscopic manuscripts. There are only 8 publications that were prospective studies. In the RAS analysis there were no randomized clinical trials as oppose to laparoscopic publications where we identified 4 manuscripts. There were more cost analysis studies in the RAS arm than laparoscopy. We looked for geographical distributions of the most cited manuscripts in order to compare centers' and their citation counts and how they transitioned from laparoscopy into RAS. We found that the majority of authors and centers that were on the top ten positions of the most cited manuscripts were different for laparoscopy and RAS. The only author who remained at the top 10 for laparoscopy and RAS was Dr. Craig Peters.

**Figure 2:**
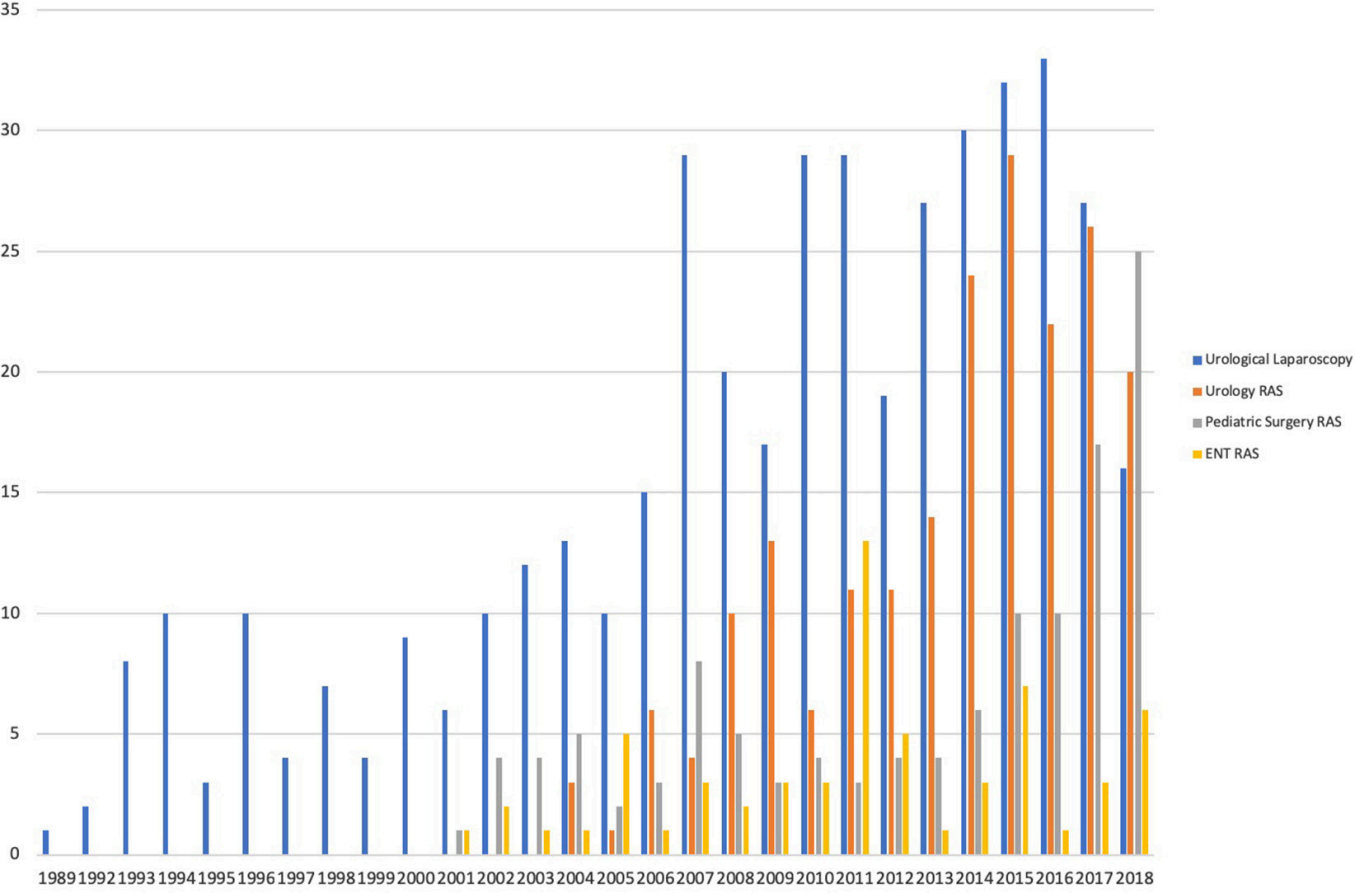
Historical count of publications for laparoscopic and robot-assisted publications.

When comparing the amount of publications per year between laparoscopic manuscripts and RAS, a higher and quicker proportional increase in the publication count for RAS manuscripts was noted (Figure 2). When comparing the amount of manuscripts published since 2,000 for pediatric urological laparoscopy, RAS urology and pediatric general surgery, the mean publication counts for this 18-year period was 20.78, 11.11, and 6.56, respectively (*p* < 0.0000) (Figure 2). When the same comparison is made for RAS in pediatric urology, pediatric general surgery and pediatric ENT there is a statistically significant difference favoring a higher productivity for pediatric urology (*p* = 0.041) (Figure 2).

In the first decade after either technique was introduced, there were less publications for laparoscopy (58 publications) than RAS (103 publications).

Average citations for laparoscopic manuscripts was 23 (*SD* ± 31) and for RAS was 20 (*SD* ± 31.5). The impact index analysis showed an average of 95 (*SD* ± 167) for laparoscopic manuscripts vs. 66 (*SD* ± 101) for RAS. When comparing average citation counts for publications before and after the year 2000 for laparoscopic procedures, the average citation counts for publications after 2000 was 20 and before 47 (CI = 17.20–35.44) (*p* < 0.0001). We also did the same comparison using the impact index scores and found an average impact index of 86 for publications after the year 2000 and 157 for those published before (*p* = 0.01).

The laparoscopic manuscript with the highest citation count had 199 citations with an impact index of 12.1. And the RAS manuscript with the highest citation count had 280 citations and an impact index of 4.3.

Our impact index analysis showed low scores for landmark papers that have remained as highly influential since their publication time in the early 2000's. Historical impact index trends show that most recent papers have more impact (Figures 4A,B).

Almost all publications came from North-America (81%) followed by Europe (15%) and the remaining from Asian and Middle-East countries. The Journal where most of the RAS manuscripts were published was the Journal of Pediatric Urology with 26.5% of the publications followed by Journal of Urology with 10.5%. For regular laparoscopy, the journal with most manuscripts was Journal of Urology with 19.2% followed by Journal of Pediatric Urology with 13.6%. By the time laparoscopy was initially introduced, the Journal of Pediatric Urology was not yet indexed.

Secondary search identified a total of 118 publications in pediatric surgery. RAS and laparoscopy have steadily increased, but pediatric surgery has not had the same proportion of increase over time as in the pediatric urology subspecialty (Figure 2).

**Figure 3:**
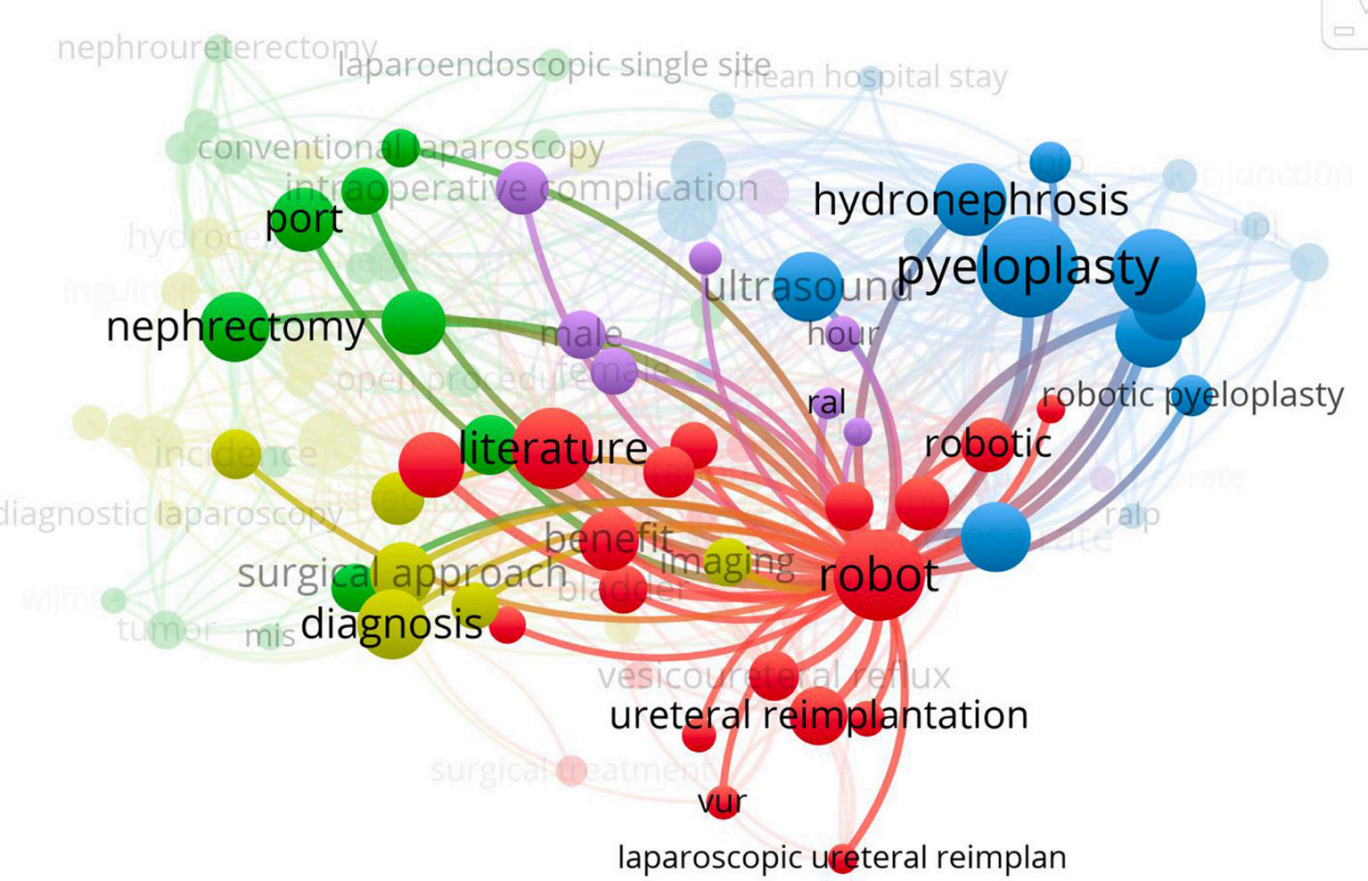
Most discussed topics for manuscripts published in laparoscopy and robot-assisted pediatric urology.

Most discussed topics were pyeloplasty followed by ureteral re-implantation (Figure 3). For Pediatric Surgery, the most common discussed topic was fundoplication. There has been a most recent increase in cost analysis publications over time for RAS. During the second decade after implementation of pediatric laparoscopy, a significant amount of manuscripts focused on the use of single port surgery. RAS literature was mainly about the presentation of applicability and safety of this technology. All publications were published exclusively in urology journals with Journal of Urology and Journal of Pediatric Urology being the ones with the most published and most cited manuscripts.

**Figure 4:**
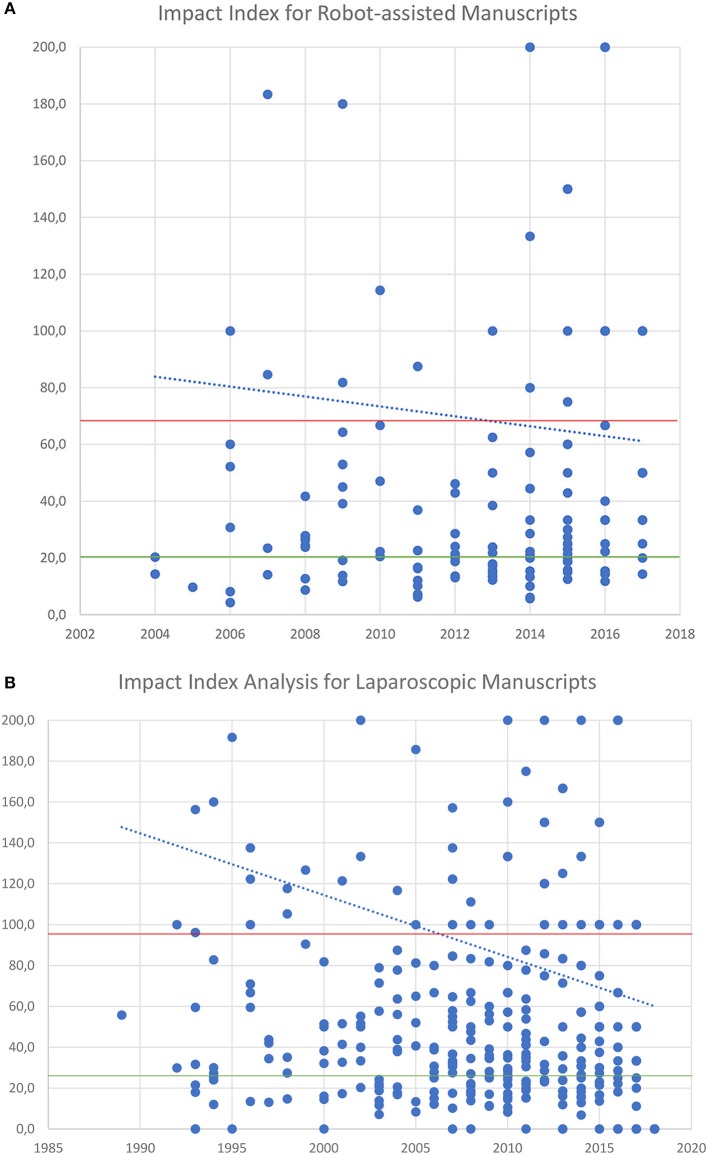
**(A)** Impact Index analysis per manuscript over time for RAS. **(B)** Impact Index analysis per manuscript over time for Laparoscopy. In red average Impact Index and green average citations per manuscript. In blue the trend of Impact index over time.

## Discussion

Minimally invasive surgery (MIS) has been a significant landmark in pediatric surgery discipline but robotic approach is evidently impacted pediatric urology evolution and practice far more than any other pediatric surgical disciplines. Our results show similar historical trends when comparing laparoscopy to RAS when looking at citation counts and level of evidence of supporting manuscripts. The main difference has been noticed on much quicker and higher manuscript productivity. Also, when adjusting by time since publication using our novel developed Impact index, we notice that RAS has had a better impact on the scientific community compared to laparoscopy. Implementation of innovative technologies depends on multiple factors and research with publications do enhance uptake of new technologies. RAS has had a quick take-off and one of the reasons for this trend is that literature acted as a catalyst for adopting it. At some degree, this supports how new technologies are accepted by the surgical community. The urge to publicize and the pressure of community for recognition, may affect the quality of scientific productivity. Our results along with other authors like Cundy et al. show that 90% of published manuscripts in this topic are level IV (3, 17). Nevertheless, careful interpretation of literature is needed when new technologies are being implemented. (18, 19). Interestingly despite finding that there are 4 randomized studies and 2 meta-analysis in the laparoscopic literature, these have not been highly cited.

In the early 1990's, the implementation of laparoscopy as a novel technology, showed to be safe and reproducible when compared to open surgery for most of the procedures (20). The possibility of internet influencing the amount of citations is plausible when analyzing the impact index for publications before and after the year 2000. A similar trend has been seen for RAS. Nonetheless, none of either technology has enabled the creation of a novel surgical technique. Surgeons are the door of entrance for many innovative technologies. Our results support how such tool has evolved and is now broadly implemented (21).

Our results do not show the same evolution for pediatric surgery, similar to what has seen by other authors (3). Our results show that most publications focused on the applicability and safety of robotic surgery when compared to regular laparoscopy or open surgery. If considering open surgery as a “gold standard” the use of laparoscopy and RAS have shown to be comparable for procedures like pyeloplasty (22, 23). This explains why it is the most reported procedure in literature. For other procedures like nephrectomy, MIS has shown to be superior with less morbidity and shorter hospital stay (24). In the case of the management of vesicoureteral reflux, ureteral reimplantation performed laparoscopically and by RAS has now shown debatable results when compared to those of open surgery (25–27).

Bibliometrics and impact factor for published literature cannot be analyzed based only on the amount of citations. It is important to consider the specialty and discussed topic amongst other variables like time since publication at the moment of interpreting this data. Our results show how MIS in pediatric urology is a very specialized topic that is read and cited by a very selective group sub-specialists. This is proven by the fact that all manuscripts are published in urological journals for both, laparoscopy and RAS. The amount of literature produced per year has never been above 50 publications and average citation counts of 20 on our analysis compared to other topics. For instance vesicoureteral reflux, which is of more interest to other specialties besides pediatric urology, the average citation counts were 101, this confirms how selective this topic can be (13). Our impact index analysis also showed that despite having similar citation averages between laparoscopic surgery and RAS, the latter has had more impact in the community and is quickly growing with more publications in 2018 than regular laparoscopy. Trends for better impact index for more recent manuscripts might be due to the preference of readers for the most recent publications.

With the ongoing debate on the cost-effective of RAS in the pediatric population (28), we noted higher number of manuscripts that tackle this particular issue (cost analysis between RAS and regular laparoscopy). This reflects the common and significant need to justify and rationalize the use of such an expensive technology (7, 23, 25, 29).

Considering that MIS has been introduced as a new technology, its implementation was never supported by research based in basic science studies or experiments. Can we say that the implementation of novel technologies may not be necessarily supported by high quality type of studies to show its efficacy. It is our opinion that the adoption of new technology in surgery may not be because of the high quality studies *per se* but rather because it is doable and can be broadly adopted then some will adopt

Our results show that about 1% of the manuscripts describe results of experiments in animal or inanimate models. One reason for this might be the interest of authors to present information on how MIS can be clinically implemented. Nonetheless, the lack of this kind of experimental research misses the opportunity for innovation and probably for higher evidence-based literature that consolidates a safer path for the safe and efficient use of this novel tool. It is interesting to see that early adopters of RAS had the highest risk of litigation (30). But after the amount of procedures performed increased, the risks dropped. Most of the claims were due to surgical complications instead device failures from the DaVinci platform. This probably supports that novel technologies may not introduce a risk for legal actions if implemented and used in a responsible way. Good quality published literature may protect surgeons from legal actions against them.

Current efforts need to focus on the development of predictable inanimate models to help support the development of instruments that respond to the high technical demands of pediatric surgery. RAS instruments have been developed to be used on adults and pediatric surgeons have adopted these instruments into pediatric patients. The small working space is a challenge that few manuscripts have addressed and there is a need for scientific evidence to answer this question to help improve the usage of RAS in small patients (31). Literature productivity on this topic has tried to answer this question. The minimal effective volume that allows the performance of different surgical tasks without arm collision is between 125 and 130 cm^3^ (31, 32). Abdominal characteristics for a suitable abdomen have been estimated to be a pubic-xyphoid length of 15 cm and an anterior superior iliac spine of 13 cm. Patient's weight has been defined as 10 kg. Considering all this variables, appropriate age range is around the 3 years of life. Considering this, many of upper tract reconstructive cases are performed earlier in life. For this reason, it is critical to focus our research on how to improve these technical limitations. Our group is currently working on developing a predictable 3-D inanimate model that simulates our results on small animal models reducing instrument collision and abdominal wall tension and traction (data presented at the NARUS conference 2018). This kind of results will allow the development of smaller instruments and improve the usage of current robot platforms in a better way.

## Conclusions

Literature productivity in pediatric laparoscopic and robot-assisted surgery topic has quickly grown. Level of evidence literature productivity has been similar for both technologies with more impact for RAS in the community, exponentially growing at a faster pace than how laparoscopy was introduced. Current graduating generations have had a significant exposure to RAS during their adult training and for this reason we believe RAS has remained a leading topic in the pediatric urology specialty (33, 34). Future directives need to focus on increasing the level of evidence to support innovation and development of pediatric instruments.

## Author Contributions

NF proposed the idea of manuscript and did the data search and analysis. WF supervised the process of the entire manuscript and made the final corrections and elaborated the discussion.

### Conflict of Interest Statement

The authors declare that the research was conducted in the absence of any commercial or financial relationships that could be construed as a potential conflict of interest.
